# 

**Published:** 1837-01

**Authors:** 


ART. I.—1. A Further Inquiry concerning Constitutional Irritation, &c. By BENJ. TRAVERS, F.R.S. (Part II. On the Pathology of the Nervous System) 1

2. Outlines of Human Pathology. By HERBERT MAYO, F.R.S. (Chaps. V. VI. and VII. Of the Nerves, the Spinal Cord, and the Enkephalon) . . ib.

3. An Essay on the Laryngismus Stridulus, &c. By HUGH LEY, M.D. . ib.

4. Pathological and Practical Researches on the Diseases of the Brain and the Spinal Cord. By JOHN ABERCROMBIE, M.D. . . . . ib.

5. Lectures on the Nervous System, &c. By MARSHALL HALL, M.D. F.R.S. L.&E. ib.

6. Medico-Chirurgical Transactions. Vol. XIX. (1. On Serous Effusions from the Membranes and into the Ventricles of the Brain, and its Connexion with Apoplexy and other Diseases of the Brain; 2. On Hypertrophy and Atrophy of the Brain; 3. On the Cure of Ramollissement of the Brain. By J. SIMS, M.D. &c.) ib.

7. Isis Revelata: an Inquiry into the Origin, Progress, and present State of Animal Magnetism. By J. C. COLQUHOUN, Esq. Advocate, F.R.S.E. . ib.

ART. II.—1. Sur l'Homme, &c. Par A. QUETELET . . . 40

On Man, and on the Development of his Faculties, &c. . . . ib.

2. Life Tables, &c. By T. R. EDMONDS, B.A. . . . . ib.

3. Tables shewing the total Number of Persons assured in the Equitable Society from its commencement in September, 1762, to January 1st, 1829, &c. . ib.

ART. III.—Outlines of Human Pathology. By HERBERT MAYO, F.R.S. &c. . 55

ART. IV.—Die Erkenntniss und Heilung der Ohrenkrankheiten. Von Dr. KRAMER 79

The Nature and Treatment of the Diseases of the Ear. By Dr. WM. KRAMER

ART. V.—1. Principles of Pathology, &c. By JOHN MACKINTOSH, M.D. . 101

2. Principles of Pathology, and Practice of Physic. By JOHN MACKINTOSH, M.D. With Notes and Additions, by S. G. MORTON, M.D. &c. . . . ib.

ART. VI.—Mélanges de Chirurgie Pratique: Emploi de l'Eau par la Methode des Affusions; Pansemens rares, &c. Par MM. JOSSE . . . 114

Observations in Practical Surgery: On the Use of Water by the Method of Affusion; on the infrequent Dressing of Wounds, &c. By MM. JOSSE.

ART. VII.—Practical Observations on various Subjects relating to Midwifery. By JAMES HAMILTON, M.D. F.R.S.E. . . . . . . . 131

ART. VIII.—1. St. Thomas's Hospital Reports. No. III. and IV. . . 142

2. Guy's Hospital Reports. No. III. September, 1836 . . . ib.

Dr. ROOTS on Porrigo, and on Hysterical Paralysis . . . 143

Mr. WEGG's Case of Chronic Dysentery, with Clinical Remarks by Dr. ROOTS 145

Mr. WHITE and Mr. TYRRELL on Diseases of the Joints . . 146

Mr. TYRRELL's Case of Stone in the Bladder . . . . . 149

Dr. ROOTS on Pericarditis, Chronic Laryngitis, and Enuresis . . 150

Mr. KEY on the Nature and Treatment of Ganglion, Bunion, &c. . 151

Mr. KING and Sir ASTLEY COOPER on the Thyroid Gland . . 153

Sir A. COOPER's Experiments on tying the Carotid and Vertebral Arteries, &c. 154

Dr. ADDISON's Observations on the fatty Degeneration of the Liver . 155

Dr. ASHWELL on Chlorosis and its Complications . . . 156

Dr. BRIGHT on Jaundice . . . . . . . 157

ART. IX.—Transactions of the Provincial Med. and Surgical Association. Vol. IV. 159

Annual Address, by Dr. PRICHARD . . . . . . 160

Dr. NIEUWENHUYS' Report of Fevers, &c. at Amsterdam, during 1834 . 161

On the Physiology and Pathology of Intermittence. By Dr. COWAN, of Bath 162

Observations on the Effects of the Secale Cornutum. By Mr. CHAVASSE . 164

Records of Ovarian Tumours. By Dr. BARLOW . . . 165

4v 90

PAGE

ART. X.—1. On the Disease of the Hip-Joint, &c. By WM. COULSON, Esq. . 169
2. On Deformities of the Chest. By WM. COULSON, Esq. . . . . ib.
ART. XI.—The Speculum applied to the Diagnostic Treatment of the Organic Diseases of the Womb. By JOHN BALBIRNIE, A.M. . . . 175
ART. XII.—On the Antidotal Treatment of the Epidemic Cholera; with a Sketch of the Physiology of this Disease, as deduced from that of Intermittent Fever. By JOHN PARKIN, Esq. . . . . . . . . 179
2. On the Efficacy of the Carbonic Acid Gas in the Diseases of Tropical Climates; with Directions for the Treatment of the Acute and Chronic Stages of Dysentery. By JOHN PARKIN, Esq. . . . . . . . ib.
ART. XIII.—1. Zur Diagnostik der Lungen und Herzkrankheiten mittelst physicalischer Zeichen: mit besonderer Berücksichtigung der Auscultation und Percussion. Von Dr. P. J. PHILLIPP, pract. Aerzte in Berlin . . 184
On the Diagnosis of Diseases of the Lungs and Heart by means of Physical Signs, &c. By Dr. P. J. PHILLIPP, Physician in Berlin.
2. On the Diagnosis of Diseases of the Chest; based upon the Comparison of their Physical and General Signs. By W. W. GERHARD, M.D. . . . ib.
3. A Bedside Manual of Physical Diagnosis, applied to Diseases of the Lungs, Pleuræ, Heart, Vessels, Abdominal Viscera, and Uterus. By C. COWAN, M.D. ib
4. Anviisning til at kjende Lunge, og Hierte, Sygdomme ved Percussion og middelbar Auscultation. Ved Dr. Med. S. TRIER, &c. . . . ib.
A Guide to the Diagnosis of Diseases of the Lungs and Heart, by means of Percussion and Mediate Auscultation. By S. TRIER, M.D.
ART. XIV.—On Insanity; its Nature, Causes, and Cure. By W. B. NEVILLE, Esq. 189

ART. XV.— THE FOREIGN JOURNALS. No. V.

COLONIAL JOURNALS:
2. The Indian Journal of Medical and Physical Science. Edited by FREDERICK CORBYN, Esq. . . . . . . 192
3. The Jamaica Physical Journal. Edited by WM. ARNOLD, M.D. . 193
AMERICAN JOURNALS:
5. The Western Journal of the Medical and Physical Sciences. Edited by DANIEL DRAKE, M.D. . . . . . . . 195
6. The Transylvania Journal of Medicine and the associate Sciences. Edited by L. P. YANDELL, M.D. . . . . . . ib.
GERMAN JOURNALS:
11. Zeitschrift für die Staatsarzneikunde. Herausg. von A. HENKE, M.D. 196
Journal of Forensic Medicine. Edited by A. HENKE, M.D.

### PART SECOND.—Bibliographical Notices.

ART. I.—Essay on the Disorders incident to Literary Men. By W. NEWNHAM, Esq. 197

ART. II.—Handbuch der Menschlichen Anatomie. Von C. F. T. KRAUSE, M.D. 198

Manual of Human Anatomy, compiled by C. F. T. KRAUSE, M.D.

ART. III.—A Discourse on Self-limited Diseases. By JACOB BIGELOW, M.D. . 200

ART. IV.—Medical Study. By J. A. SYMONDS, M.D. . . . 201

ART. V.—On the most frequented Watering Places on the Continent. By E. LEE 202

ART. VI.—The Practical Anatomy and Elementary Physiology of the Nervous System. By F. LE GROS CLARK . . . . . . . 203

ART. VII.—Aur. Cor. Celsus on Medicine. By ALEXANDER LEE, A.M. . 204

ART. VIII.—The Botany, &c. of the Himalayan Mountains. By T. F. ROYLE, Esq. 205

ART. IX.—Practical Demonstration of the Human Skeleton. By G. ELKINGTON, Esq. ib.

ART. X.—A Medical Vocabulary. By a Medical Practitioner . . 206

ART. XI.—The British Medical Almanack for 1837. Edited by WM. FARR . ib.

ART. XII.—Cataract: its Nature, Symptoms, &c. By JOHN STEVENSON, Esq. 207

ART. XIII.—An Address delivered at the Fourth Anniversary Meeting of the Medical and Surgical Association, July 20, 1836. By J. G. CROSSE, Esq. . 208

### PART THIRD.—Selections from Foreign Journals.

PAGE

ANATOMY, PHYSIOLOGY, AND PATHOLOGY.

On the Theory of Congestion, Hemorrhage, Plethora, &c. By Prof. NAUMANN 209

On Gastroperiodynia. By THOMAS A. WISE, M.D. . . . 215

On the Oxide of Bismuth in Yellow Fever. By J. MAGRATH, Esq. . . 217

On the Cachexia Africanorum. By JOHN FERGUSON, M.D. . . ib.

On Milk-Sickness. By Drs. SHELTON, WHITE, and MACANELLY . . 218

On certain Pathological Changes in the Arterial System. By M. BIZOT . 220

Pathological Observations. By J. F. H. ALBERS, M.D. . . . ib.

Effect of repeated Loss of Blood on the Constitution of Blood-vessels. By Dr. NASSE 221

Communication of Spinal Abscess with the Bronchi. By Dr. STANNIUS . 222

PRACTICAL MEDICINE AND THERAPEUTICS.

On Debility in Nervous Diseases, and on Tonics in Insanity. By Dr. GUISLAIN 223
On the Diagnostic Value of the Deformities of the Chest, &c. By M. WOILLEZ 226
History of a Deaf and Dumb Person; with Dissection. By Dr. BERGMANN . 227
Singular Case of Inflammation of the Muscles. By J. A. PETET . . ib.
On Hypertrophy, with Dilatation of the Heart, in Children. By Dr. TOEL . 228
On the Therapeutic Value of Extracts of the Solaneæ containing Green Fecula. By MM. MARTIN-SOLON and SOUBERAIN . . . . . 229
Tic Douleureux cured by Tartrate of Antimony. By Dr. HAUSBRANT . . 230
On Sulphate of Quinine introduced into the Nostrils in Iralgia. By M. ST. HILAIRE ib.
Cure for Drunkenness . . . . . . 231
Stuttering occasioned by Worms. By Dr. SCHULTZE . . . ib.

SURGERY.

On Neuralgias of the Urethra and Neck of the Bladder. By M. CIVIALE, D.M.P. ib.
On the Section of the Tendo Achillis in the Treatment of Club-feet. By M. BOUVIER 233
On the Radical Cure of Hernia in America . . . . 234
Cases of Foreign Bodies introduced into the Stomach and Anus. By J. P. DOR . 236
Statistical Results of Amputations. By M. A. GENDRON . . . 237
On Inguino-interstitial Hernia. By G. GOYRAND, D.M. . . . ib.
Remarks on some of the remote Effects of Fractures of the Femur. By Dr. GUYOT 238
On Inflammation of the Mucous Glands of the Male Urethra. By Dr. KLEEBERG 239
On the Elevation and Depression of the Pelvis, in Luxations of the Femur; and on certain Forms of Lameness, hitherto undescribed. By JULES GUERIN, M.D. . 240
On a new Mode of treating Herpes, (Dartres.) By Dr. BUGLIARELLI . 241
New Method of reducing Luxations of the Humerus. By M. GERARD . ib.
New Treatment of Strictures of the Urethra. By M. JOBERT . . 242
Obliteration of the Brachial Artery by Compression of a Pad. By J. P. DOR . ib.
On Sulphuret of Lime in Diseases of the Skin. By Dr. SAVARDAN . . ib.

MIDWIFERY.

A Case of Spontaneous Version. By Dr. FR. NIESS . . . 243
On Injections through the Umbilical Vein in retained Placenta. By Dr. ALBERT 244
Tubal Pregnancy, ending in Rupture of the Tube. By Professor BUSCH . 245
On Putrescence of the Uterus. By Dr. ALBERT . . . . 246
On the Signs of Pregnancy. By Dr. OSIANDER . . . . 247
Successful Cæsarean Operation from an accidental Wound. By N. E. PIGNÉ . 248

FORENSIC MEDICINE.

Case of Death from violent Blows. By Professor VON SIEBOLD . . ib.

HYGIENE.

Cases of Death and Disease by unwholesome Dwellings, &c. By M. D'ARCET, &c. 252

MEDICAL STATISTICS.

Statistics of Calculus in Austria . . . . . . 254
Medico-Statistical Report of the Deaf and Dumb in Brunswick. By Dr. MANSFELD 255

VETERINARY MEDICINE.

On the Influence of the Epidemic Cholera Constitution upon Animals . . 256

### PART FOURTH.—Selections from British Journals.

PAGE

### ANATOMY, PHYSIOLOGY, AND PATHOLOGICAL ANATOMY.

On the Anatomy of the Fifth Nerve. By B. ALCOCK, M.B. . . . 257
On the Mechanism of Bruit de Soufflet, or Bellows-Sound. By D. J. CORRIGAN, M.D. ib.
On the Vital or Self-moving Powers inherent in the Blood. By R. M. HAWLEY, M.D. 258
Determination of the Question, which are the Nerves of Taste? By B. ALCOCK, M.B. 259
On the Hemorrhagic Hepatization of the Lungs. By R. KNOX, M.D. . . ib.

### PRACTICAL MEDICINE AND THERAPEUTICS.

On Typhus Fever in Britain compared with that in Paris and Geneva. By Dr. LOMBARD 260
Case of Spectral Illusions, with Loss of the Memory of Words. By J. CRAIG, Esq. 261
On Dropsy following Scarlet Fever. By J. STARK, M.D. . . . 262
On distinguishing Neuralgic from Inflammatory Affections. By W. GRIFFIN, M.D. ib.
Effects of the Saline Treatment in Morbid Conditions of the Blood. By C. R. BREE, Esq. 263
Cases of Spasm of the Muscles of the Neck, &c. By R. HUTCHINSON, M.D. . ib.
On the Nature and Treatment of Acute Neuralgia. By F. C. SKEY, Esq. . ib.
BARON SLOET's Method of treating Epilepsy . . . . ib.
Influence of Mental on Bodily Functions. By C. O'REILLY, M.D. . . 264
On the Use and Abuse of Aloes. By E. GREENHOW, M.D. . . . ib.
ST. JOHN LONG's Liniment . . . . . . . ib.

### SURGERY.

On the Treatment of Vascular Nævi by Injection. By E. A. LLOYD, Esq. . 265
On anomalous Affections of the Larynx requiring Tracheotomy. By W. H. PORTER, Esq. ib.
Case in which a Pebble remained eight weeks in the Larynx. By H. BULLOCK, Esq. ib.
On Extracting Foreign Bodies from the Ear. By N. HILL, M.D. . . 266
On Lithotrity as practised by the late PHILIP FERNANDEZ, Esq. . . ib.
Aneurism of the Arteria Innominata successfully treated. By S. W. FEARN, Esq. ib.
On Amputation of the Penis. By J. MORRISON, M.D. . . . ib.
New Treatment of Onychia Maligna. By C. RAY, Esq. . . . ib.

### MIDWIFERY.

On Binding the Abdomen of Lying-in Women. By HUGH LEY, M.D. . 267

On spontaneous Amputation of the Limbs of the Fœtus in Utero. By Dr. SIMPSON ib.

### MEDICAL STATISTICS.

Statistics of Fever in Belfast. By W. MATEER, M.D. . . . 268
Comparative Frequency of the principal Medicines prescribed, &c. By Dr. MOORE 269
Statistics of Suicide in Aberdeen. By F. OGSTON, M.D. . . . 270
Statistics of the London Hospital, &c. By T. R. EDMONDS, Esq. . . ib.

### FORENSIC MEDICINE.

Account of a Trial, which occurred at the Perth Assizes . . . 271

### CHEMISTRY.

New Method of preparing Iodic Acid. By LEWIS THOMPSON, Esq. . . 272

### TOXICOLOGY.

Deleterious Effects of Pork. By J. M'DIVITT, M.D. . . . ib.

### PART FIFTH.—Medical Intelligence.

On the PRESENT STATE OF MEDICINE in the UNITED STATES, (continued) . 276
Scale of Medical Fees for the Kingdom of Bavaria . . . 282
Prize Essays for 1837-8 . . . . . . . 287
Homœopathy . . . . . . . . 288
The British Medical Association . . . . . . 289
Provincial Medical and Surgical Association. District Branches . . 290
Prize Medals granted by the Royal Society . . . . 291
Medical Graduations at Edinburgh, 1836 . . . . 292
Diplomas and Certificates granted at Edinburgh and London, 1835-6 . ib.
New Regulations respecting Teachers of Anatomy, &c. . . . ib.
Prize Medals of the Apothecaries' Company and the College of Surgeons . 293
CHARTER of the LONDON UNIVERSITY . . . . . ib.
OBITUARY—Dr. BOISSEAU . . . . . . . 297
BOOKS RECEIVED FOR REVIEW . . . . . . 298

